# Policy Requirements for the Prevention and Management of Fetal Alcohol Spectrum Disorder in South Africa: A Policy Brief

**DOI:** 10.3389/fpubh.2021.592726

**Published:** 2021-04-15

**Authors:** Babatope O. Adebiyi, Ferdinand C. Mukumbang, Anna-Marie Beytell

**Affiliations:** ^1^Centre for Interdisciplinary Studies of Children, Families and Society, University of the Western Cape, Cape Town, South Africa; ^2^School of Public Health, University of the Western Cape, Cape Town, South Africa; ^3^Department of Social Work, University of the Western Cape, Cape Town, South Africa

**Keywords:** fetal alcohol spectrum disorder, policy, guideline, prevention, management, South Africa, policymakers, service providers

## Abstract

This policy brief is aimed to guide policymakers in developing a comprehensive and multi-sectoral policy for the prevention and management of fetal alcohol spectrum disorder (FASD). FASD is a leading source of non-genetic developmental and intellectual disability globally and is usually associated with primary and secondary disabilities. South Africa has been identified to have the highest reported prevalence of FASD in the world. Nevertheless, evidence shows that there is no specific policy for FASD, albeit there are clauses that could be attributed to its prevention and management in other existing policies. In this brief, we present a guideline to inform programmes and interventions to tackle the FASD problem in South Africa and other relevant contexts through developing a policy.

## Summary of Findings

There is no specific policy for FASD in South Africa, however, there are clauses in other relevant South African policy documents that could be attributed to the prevention and management of FASD.

Awareness and education on the dangers of consuming alcohol during pregnancy and assisting individuals with alcohol problems are some of the strategies reported for the effective prevention of FASD.

Management strategies include routine screening of babies confirmed to have been exposed to alcohol during pregnancy and training of teachers on classroom management.

Findings from this study promote the attainment of goals three and four of Sustainable Development Goals by improving the quality of life for individuals, thereby enhancing the intellectual capabilities of individuals to attain the highest possible level of education.

## Introduction

Fetal alcohol spectrum disorder [FASD] is a diagnostic term describing a range of conditions affecting persons exposed to alcohol during pregnancy (1). These conditions can be classified under four groups, i.e., fetal alcohol syndrome [FAS], partial FAS, alcohol-related neurodevelopmental disorders and alcohol-related birth defects (2). According to the Center for Disease Control and Prevention and the U.S. Surgeon General, there is no known safe amount and no known safe time during pregnancy to consume alcohol (3).

According to the World Health Organization, South Africa has the highest reported per capita rates of alcohol consumption in the world among those who do consume alcohol (4, 5). It is widely believed that heavy drinking among poorer South Africans is deeply-rooted in the legacy of the “dop” system, whereby alcoholic beverages were offered to farmworkers as part of their wages (6). Alcohol consumption during pregnancy is also widespread in South Africa at a rate ranging from 2.5 to 45% (7). The above-mentioned facts could explain why South Africa is considered to have the highest reported prevalence of FASD in the world, which ranges from 29 to 290 per 1 000 live births (8).

FASD may lead to primary disabilities such as intellectual disability, learning difficulties, poor impulse control, problems with attention, memory loss, social perception, reasoning and using judgement, cognitive processing, mathematics and language deficits, and developmental lags (9). Some secondary disabilities also associated with FASD include mental health problems, disrupted school experience, trouble with the law, custody, inappropriate sexual behavior, alcohol/drug problems (9).

Despite being a preventable disorder, according to the Foundation for Alcohol Related Research [FARR], about seven million people living in South Africa exhibit characteristics associated with FASD (10). FARR reported that FASD has both financial and social implications for individuals, families and society with government and taxpayers bearing the greatest burden. FARR also indicated that the cost of FASD in South Africa can be comparable to that of the USA [$6 billion—approximately R42 billion annually]. The current services and interventions to address FASD are fragmented across relevant departments at national and provincial levels due to the lack of multisectoral policy and inclusive policy implementation (11, 12). In addition, the “best buys” [increasing excise taxes on alcoholic beverages, comprehensive restrictions on alcohol advertising, and restrictions on sales of alcohol] for reducing harm from alcohol have not been implemented in South Africa (13–15).

Furthermore, there is a need for the decolonisation and re-contextualization of the current policy discourse (16). The proponents of the decolonised policy discourse believe that women should be seen as victims of the FASD problem, not as perpetrators. They also propose that the socio-economic and socio- political circumstances that predispose women to alcohol consumption during pregnancy, which may lead to FASD, need to be addressed (16). Also, considering the huge financial and social implications of FASD, there is a need to develop an effective policy for FASD. The development of an effective policy for FASD could also facilitate the achievement of South African National Development Plan [NDP]—Vision 2030 (17), which aims to eliminate poverty and reduce inequality by 2030. This policy brief highlights policy requirements for the prevention and management of FASD in South Africa.

## Policy Options and Implications

### Method

**Figure 1:**
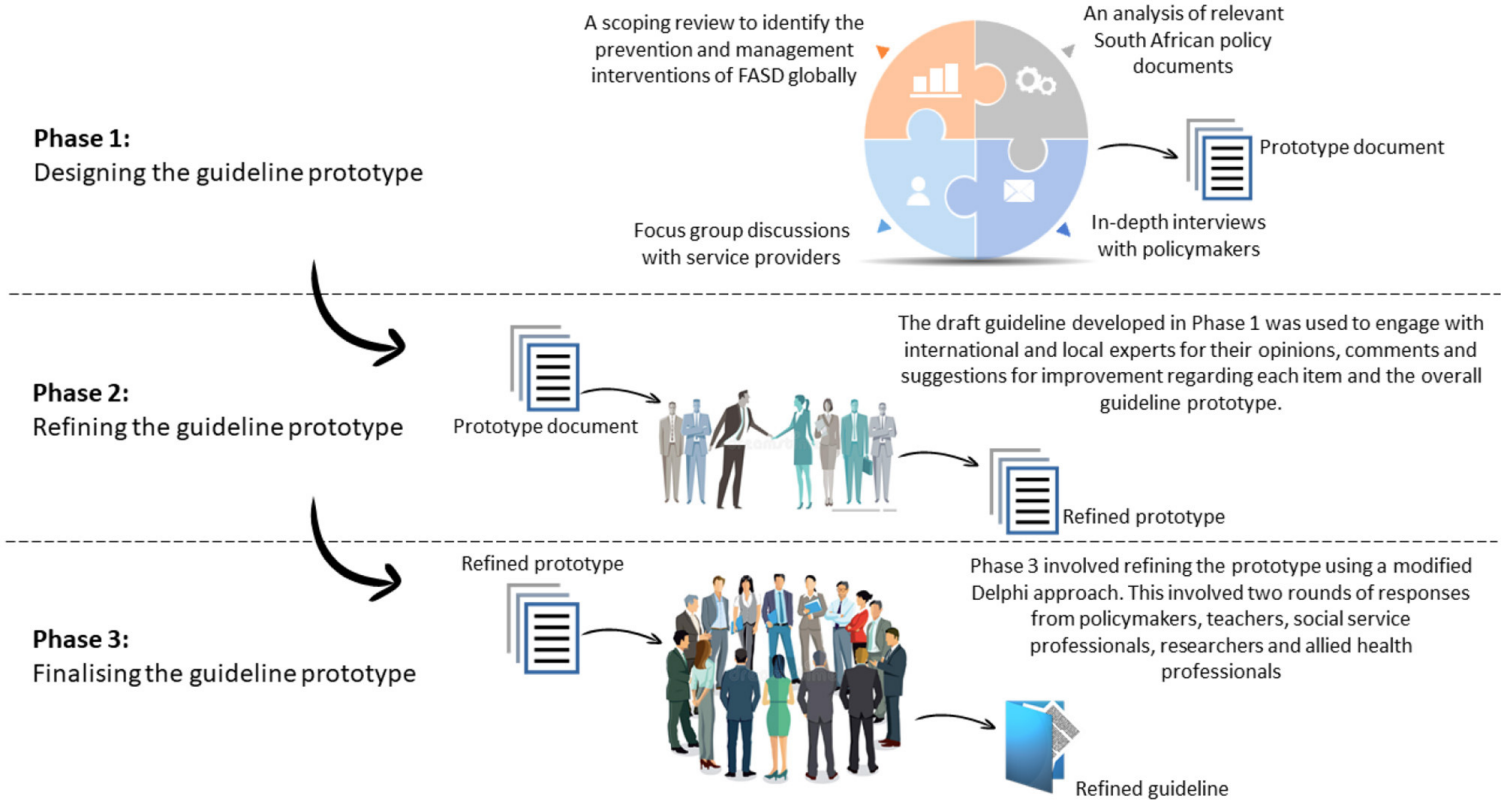
Study design approach.

We adapted the WHO's approach to guideline development (18, 19). The study was conducted in three phases (Figure 1) (20). In the first phase, two qualitative studies were conducted (in-depth interviews with policymakers and focus group discussions with service providers). Participants were asked about the policy requirements for the prevention and management of FASD. In addition to the qualitative studies, a document review of relevant South African policy documents was conducted. Furthermore, we conducted a scoping review of the prevention and management interventions for FASD globally. The findings from these studies were aggregated to develop an initial guideline prototype. In Phase 2, local and international experts were consulted to refine the initial prototype developed in phase 1. Phase 3 involved engaging with experts on FASD through a two-round Delphi process to develop the final guideline prototype.

### Findings

Findings from the two qualitative studies (21, 22) and a document review (23), which formed part of our study, confirmed that there is no specific policy document addressing FASD in South Africa. However, clauses that could be attributed to the prevention and management of FASD exist in other related policy documents, although these do not holistically address FASD. The participants enumerated the guiding principles and approaches for policy development and the policy requirements for the prevention and management of FASD in South Africa. Also, findings from a scoping review (24) indicated the availability of evidence-based interventions for the prevention and management of FASD.

In the Delphi study (20), the experts identified measures that could be included for the prevention and management of FASD in South Africa based on the refined prototype. Some of the guiding principles and approaches include collaboration, consider the family as a unit, human right-based, holistic, evidence-based, culturally diverse and sensitive, considering input from individuals with FASD and their families and addressed social determinant of health. The identified policy requirements for the prevention of FASD comprised of awareness and education on the dangers of drinking alcohol during pregnancy, promotion of the use of contraceptives, skills development and social programmes and assisting individuals with alcohol problems. Routine screening of babies, training of professionals, skills development and support for individuals with FASD were some of the identified policy requirements for the management of FASD.

### Actionable Recommendations

In developing the policy for FASD, we recommend the following based on available and relevant evidence.

#### Guiding Principles

The policy should incorporate the following guiding principles. Policy development to prevent and manage FASD should be multi-sectoral—a collaboration between different sectors [departments]. The consequences that may arise from prenatal alcohol exposure, usually encompassing medical, educational and social problems warrant the collaboration of the relevant departments, which currently work in silos. Therefore, to address FASD holistically, departments such as Education, Health, Social Development, Labor, Trade and Industry, Justice and Correctional Services should collaboratively work together.

Social determinants of health such as socioeconomic status, living conditions, level of education contribute significantly to the increase in the prevalence of FASD. There is a need for FASD prevention and management interventions to target these social determinants. Furthermore, interventions for prevention and management must be based on current evidence and culturally diverse and population-sensitive. Additionally, a family-centered approach should be considered in the development of the policy. This family-centered approach encourages input from individuals with FASD and their families and ensures their human rights are protected. Moreover, there is a need to decolonise the current policy approach; women should be victims and not perpetrators of the problem.

#### Requirements for the Prevention of FASD

FASD is preventable, therefore, there is a need to develop holistic prevention strategies to reduce the prevalence of FASD and the number of individuals who will require management services. There is a need to consider the implementation of the ‘’best buys” for reducing harm from alcohol [pricing policies and restrictions to alcohol availability and marketing]. In addition, there is a need for awareness and education on the dangers of drinking alcohol during pregnancy in communities, health facilities and schools, targeting adolescents. Awareness and education should also target the general population.

Because unplanned pregnancies contribute to the increase in the prevalence of FASD. To reduce the number of unplanned pregnancies, families should be encouraged to use various effective family planning approaches. Furthermore, individuals with alcohol problems should be assisted to access and complete treatment and supported to integrate into the society after treatment. Promoting skills development and other social programmes are also necessary to engage individuals with alcohol problems. Also, relevant service providers should be trained to be able to document and counsel individuals appropriately as well as pregnant women with alcohol problems.

#### Requirements for the Management of FASD

The consequences of prenatal alcohol exposure may lead to both primary and secondary disabilities. A comprehensive strategy is required to manage these disabilities timeously to improve the quality of life for individuals with FASD. FASD-related screening of babies with confirmed exposure to alcohol during pregnancy should be routinised. Routinised screening can promote early diagnosis and intervention to prevent secondary disabilities. The availability of relevant service providers to support the routine screening of prenatal alcohol-exposed children is necessary as it requires a multidisciplinary team of professionals to successfully diagnose FASD. Early diagnosis and the establishment of national surveillance for FASD are encouraged to create a mechanism for managing FASD. Training of teachers on classroom management should also be included in an FASD policy as individuals living with FASD may find the standard classroom environment challenging and may require additional support to cope.

There is a need to consider enacting an FASD policy that promotes school of skills [schools where pupils are taught skills that will enable them to enter the labor market] and special schools for individuals with FASD who are not benefiting from the mainstream schools because of their low intellectual abilities. When designing FASD programmes and interventions, support for individuals with the disorder and their biological parents and caretakers should also be considered.

## Conclusions

FASD is a lifelong disability that affects individuals, families and communities, preventing people from actualising their potentials, thus it is recognized as a public health problem. However, there is no specific policy to guide prevention and management efforts of FASD in South Africa, where there has been a persistent increase in FASD prevalence despite current prevention efforts. Consequently, there is a need for the decolonisation—without looking at how varying privilege, power and socialization of different genders and ethnic groups impacts on peoples—of the current FASD prevention and management approaches to improve their effectiveness. To achieve Vision 2030 [National Development Plan], strategies for the prevention and management of FASD should be streamlined within the current integrated services.

## Author Contributions

BA, FM, and A-MB conceived and drafted the outline of the manuscript. BA and FM acquired and interpreted the information gathered for the work. BA, FM, and A-MB provided critical input on implications and recommendations. BA drafted sections of the manuscript. All authors contributed to manuscript revision, read, and approved the submitted version.

## Conflict of Interest

The authors declare that the research was conducted in the absence of any commercial or financial relationships that could be construed as a potential conflict of interest.
